# An Ethical Framework for the Design, Development, Implementation, and Assessment of Drones Used in Public Healthcare

**DOI:** 10.1007/s11948-020-00233-1

**Published:** 2020-06-23

**Authors:** Dylan Cawthorne, Aimee Robbins-van Wynsberghe

**Affiliations:** 1 The Faculty of Engineering, Drone Center, Mærsk Mc-Kinney Møller Institute, University of Southern Denmark, Campusvej 55, 5230 Odense M, Denmark; 2 Ethics/Philosophy of Technology Section, Department of Values, Technology and Innovation, Faculty of Technology, Policy and Management, Delft University of Technology, 2600 AA Delft, The Netherlands

**Keywords:** Robot ethics, Value-sensitive design (VSD), Values hierarchy, Applied ethics, Public healthcare, Drones

## Abstract

The use of drones in public healthcare is suggested as a means to improve efficiency under constrained resources and personnel. This paper begins by framing drones in healthcare as a social experiment where ethical guidelines are needed to protect those impacted while fully realizing the benefits the technology offers. Then we propose an ethical framework to facilitate the design, development, implementation, and assessment of drones used in public healthcare. Given the healthcare context, we structure the framework according to the four bioethics principles: beneficence, non-maleficence, autonomy, and justice, plus a fifth principle from artificial intelligence ethics: explicability. These principles are abstract which makes operationalization a challenge; therefore, we suggest an approach of translation according to a values hierarchy whereby the top-level ethical principles are translated into relevant human values within the domain. The resulting framework is an applied ethics tool that facilitates awareness of relevant ethical issues during the design, development, implementation, and assessment of drones in public healthcare.

## Introduction

In recent years the number, capabilities, and applications of non-military drones have grown exponentially, surpassing military use in 2013 (Choi-Fitzpatrick et al. 2016). Drones are flying robots—unoccupied aircraft that can fly at some level of autonomy (Villasenor 2012) and reliably sustain flight in order to perform useful functions (Clarke 2014b). Hobbyists, activists, journalists, film makers, humanitarian organizations, and inspection agencies are all exploring the possibilities for drones to realize their interests, and all have been confronted with the societal risks involved. Drones can give people “eyes” in places they might not otherwise be able to reach, e.g. activists wishing to observe the maltreatment of animals behind high fences impossible to climb (Taylor 2019) or to observe protests to report in journalism (McKay 2019).

The use of drones in humanitarian contexts is especially enticing in areas with limited infrastructure and challenging terrain (Cawthorne and Cenci 2019; Meier et al. 2017; USAID 2017; van Wynsberghe and Comes 2019). Future applications of drones in healthcare include delivering items such as blood samples, medications, vaccines, and organs, between healthcare institutions and directly to patients’ homes (Rosser et al. 2018). Drones have even been suggested for indoor use to deliver medications right to patients’ bedsides (Tucker 2017).

Now, public health institutions in developed countries are looking to the possibility of using drones to provide more rapid and cost-effective healthcare in light of dwindling healthcare resources and personnel (Knoblauch et al. 2019). Enshrined in the healthcare tradition are codes of conduct and values of wellbeing that must structure the introduction of any new drug, practice, or technology. The question then is how to ensure that drones conform to this standard. How should the design, development, implementation, use, and assessment of this new technology adhere to the moral codes found within healthcare?

In the field of robot ethics, there are a variety of reflections on the use of robots in the healthcare space to assess the impact on patient’s privacy (Sharkey and Sharkey 2012), human rights (Sharkey and Sharkey 2011), and autonomy (Sparrow 2016). Academics have also addressed the impact of robots on healthcare providers (Vallor 2011; van Wynsberghe and Li 2019) and on the overall care provision, referred to as the care practice (Santoni de Sio and van Wynsberghe 2016; van Wynsberghe 2012, 2013a, 2016). Moving such reflections forward, it is time to bring this moral framework into the design and development of public healthcare drones—in a way that supports the engineers and designers creating them, and in a way that ensures the timely reflection of ethical issues prior to their ubiquitous use.

We suggest that the construct of an ethical framework provides an accessible approach for designers to engage with ethical issues in need of attention. However, the ethical issues stated above do not specifically target a healthcare domain, or a new technology introduced into this domain. Alternatively, one could look for approaches directly from robot ethics that focuses on the impact of introducing a technology into a practice (e.g. care-centered value sensitive design, or CCVSD van Wynsberghe 2012, 2013a, b, 2016), the use of the human–robot interaction HRI model to guide the evaluation (van Wynsberghe forthcoming), or the impact of the drone on the overall healthcare system (e.g. human–robot-system interaction or HRSI van Wynsberghe and Li 2019). Each provides a distinct vantage point, yet what is missing in the literature is a framework specific to the drone developer or implementer to provide tailored guidance.

For example, the CCVSD approach, and more specifically the nature-of-activities approach (Santoni de Sio and van Wynsberghe 2016) can be used to assess the activities drones will perform, and “certain practice-oriented activities in healthcare should arguably be left to humans, but certain (predominantly) goal-directed activities in healthcare can be fulfilled (sometimes even more ethically) with the assistance of a robot” (Santoni de Sio and van Wynsberghe 2016). The ethical framework approach addresses the use of drones in practices where the drone fills an instrumental (goal-directed) need for the doctors and clinicians, and there is no direct interaction with the patient. An example is transportation of medical supplies and samples between hospitals. However, if interaction with the patient is required, this task might be best analyzed using CCVSD, and be performed by a human (Santoni de Sio and van Wynsberghe 2016), such as a drone that brings medications directly to a patient at their home. Here, the patient interacts directly with the drone rather than with the caregiver, and there is a risk of devaluing the care practice.

In the following paper we frame drones in healthcare as a social experiment. The concept of ‘technology as a social experiment’ is defined as one where “only limited operational experience” exists, and benefits and risks cannot easily be assessed based on experience (van de Poel 2016). This requires a proactive ethical approach to guide the research and development of drones used instrumentally in public health, due to the emergent nature of their implementation, and the high stakes.

We propose a framework to ethically evaluate and proactively guide the design of drones in healthcare contexts by using the bioethics principles as the foundation: beneficence, non-maleficence, autonomy, and justice (Beauchamp and Childress 2001). Drones possess features of artificial intelligence (AI), and therefore we add a fifth ethical principle: explicability (Floridi et al. 2018). Paying tribute to the abstract nature of these principles and the need to translate principles into actionable design requirements, we suggest contextually relevant values that can be operationalized in the design, development, implementation, and assessment of the technology.

The ethical framework for drones in public healthcare is built using the starting point of value sensitive design (VSD)—that values should be explicitly included into the design of new technology—coupled with the translation of values into norms as presented by van de Poel (2013). The framework can, for example, be utilized in the conceptual investigation stage of a VSD process (Friedman et al. 2013) and help to surface relevant ethical concerns and potential social impacts of the technology. The ethical framework is not meant as a stand-alone checklist; rather, it is meant as a starting point for ethical reflection in technology development which can be used for multiple iterations of a design, as VSD prescribes. The framework is an applied ethics tool, intended to structure concerns and opportunities that designers and implementers should be paying attention to, and working to mitigate or enhance, respectively, when possible.

Although drones have been in operation for several decades, there is still little experience with drones in public spaces; in fact, most operational experience comes from war zones and humanitarian contexts. In framing drones in public healthcare as a social experiment taking place in society, engineers and designers must proceed cautiously and within proper ethical constraints and epistemic goals. Ethical constraints include that the technology be used only when its use does not increase risk to vulnerable demographics. Epistemic goals refer to having a clear hypothesis about the specific ways people might benefit from the technology in question—something to be studied, rather than using technology for technology’s sake.

Utilizing an ethical framework can help determine the acceptability of a technology as it is unfolding rather than having to attempt to foresee all the risks beforehand (van de Poel 2016). As Palm and Hansson point out “predicting the future of a technology is a vain undertaking with low chances of success” (2006). What’s more, if technology development and implementation proceed with the assumption that all (or most) risks have been thought about beforehand, there will be no mechanisms in place to uncover new or unintended risks in parallel with the technology’s development or use.

Currently, there is no existing ethical framework for designers of drones in public healthcare. Therefore, we propose to fill this gap by creating a framework and suggest it would be beneficial to adopt. Given the criticality of the context and the role that the bioethics principles play in structuring the healthcare domain, they should also play an integral role in the evaluation of drones (or any other robot for that matter) used in healthcare contexts and practices. Still, more detail will be needed if the principles are to be used to structure an ethical framework for drones in public health. To achieve this, we suggest the values hierarchy approach of van de Poel, which demonstrates the translation of abstract values into contextual norms, and then into specific, actionable design requirements (van de Poel 2013).

## Value Sensitive Design and Values Hierarchy

In framing drones in healthcare as a social experiment, we recognize that ethical guidelines are needed to protect subjects involved in the experiment. We suggest the ethical framework as an appropriate ethical constraint, and that the creation of overarching ethical constraints adhere to the four bioethics principles (Beauchamp and Childress 2001) and the AI ethics principle of explicability (Floridi et al. 2018). To be sure, the bioethics principles of have been hotly debated since their introduction due to their lack of concrete guidance to practitioners in the healthcare space. They are meant as a starting point to uncover ethical issues and as a tool for debate when ethical issues arise, rather than a silver bullet to end all ethical discussions. Given the abstract nature of these principles we suggest the concept of ‘translation and operationalization’ in the form of the values hierarchy (van de Poel 2013) to facilitate the translation of values into concrete and actionable design requirements.

Beginning from the theory of value sensitive design (VSD), one is encouraged to surface and proactively support certain (human) values via product design and specification. The values hierarchy builds on this, but works to facilitate the translation of (abstract) human values into (tangible) design requirements and, conversely, to demonstrate how technical design requirements can support or diminish certain human values.

**Fig. 1:**
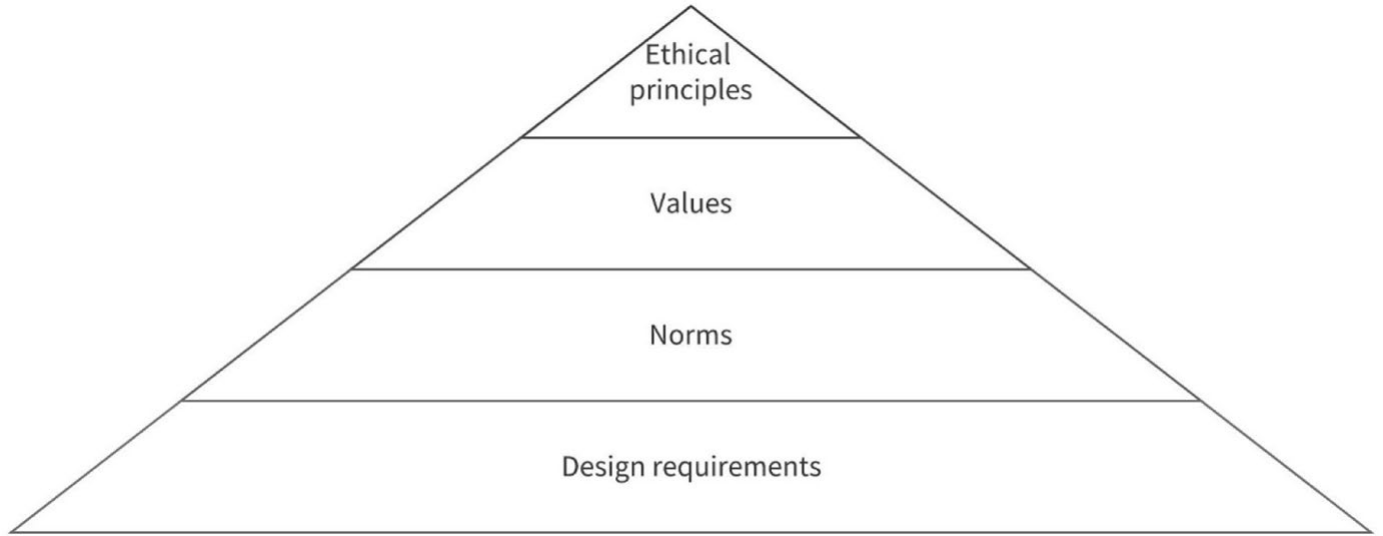
The values hierarchy in this work consists of ethical principles, human values, norms, and design requirements (graphic by the authors, inspired by (van de Poel 2013))

In this work, the values hierarchy contains four levels, as shown in Fig. 1: ethical principles, human values, norms, and design requirements. Ethical principles constitute the top of the hierarchy and represent the most abstract and far-reaching principles we wish to uphold. They have intrinsic (rather than extrinsic) value and should be pursued for their own sake (van de Poel 2013). The ethical principles from bioethics are utilized: beneficence, non-maleficence, human autonomy, justice (Beauchamp and Childress 2001). It is important to note that the drone will have a variety of appearances and capabilities, one of which may be the inclusion of artificial intelligence (AI). Because of this possibility we expand the list of principles from four to five, to include the principle of explicability (Floridi and Cowls 2019; Floridi et al. 2018). These ethical principles are non-exhaustive—it is possible that some ethical concepts have been overlooked; the principles are overlapping—human autonomy can also be considered beneficial; and the principles are normative—for example, many claim that human autonomy is a good thing.

The ethical principles are abstract and in need of further contextualization and specification; therefore, next in the values hierarchy are the human values to be supported. Values in the context of engineering design can be conceptualized in several ways, including economic, utility, moral, cultural, and aesthetic value (van de Poel 2009). In VSD we are most interested in “human values with ethical import” (Friedman et al. 2002). If we consider the principle of beneficence, a relevant human value may be privacy such that people do not feel they are being watched by the healthcare drone.

Values can and often do come into conflict in real-world technology development (Cawthorne and Wynsberghe 2019; Cuppen et al. 2016), and many values may be incommensurable (not directly comparable) with each other. Negotiating these conflicts is a critical part of engineering and design, and there are several approaches to dealing with them including: using direct trade-off when values are commensurable, using innovation to eliminate the conflict altogether, or “satisficing with moral obligations”, where morally-acceptable thresholds are set for the relevant values (van de Poel 2015).

Beneath the values level in the values hierarchy are norms, which contain “prescriptions for, and restrictions on, action”. Common end-norms (capabilities to be achieved) in engineering are design objectives, such as ‘maximize safety’ or ‘minimize costs’ (van de Poel 2013). Norms situate the values within the relevant context of use. For example, there are norms, cultural ideals, and laws around the value of privacy in public spaces that healthcare drones will fly over, and these vary depending on the context or country the drone is operating in.

At the base of the values hierarchy are design requirements. In VSD, this is where the relevant principles, values, and norms are ‘built in’ to the product. In the earlier example of a healthcare drone operating over public spaces, and given the desire to maintain people’s privacy, the relevant design requirement could be that the drone does not use a camera for precision landing. Instead, it uses ultrasonic (sonar) sensors which cannot capture personal data. Or, if a camera must be used, the system includes anonymous video analytics software to ensure ‘privacy-by-design’ (Cavoukian 2012) which will be discussed later in the section on capability caution.

**Fig. 2:**
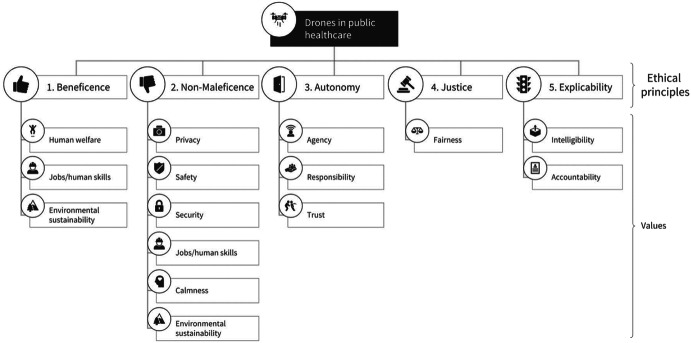
The two top levels of the values hierarchy for drones in public healthcare, including ethical principles and human values. Practitioners must translate these into contextual norms, and then design requirements based on the specific use-case. The framework is meant as a starting point for ethical reflection in the development of healthcare drones that fill an instrumental (goal-directed) need. It should not be applied in an overly-rigid manner; instead, it should provide a framework of concerns and opportunities for engineers and designers to consider when developing drone technology (graphic by the authors)

In what follows, we propose in detail the upper two levels of the hierarchy—the ethical principles and relevant human values within the healthcare domain, as shown in Fig. 2. Then, we discuss how practitioners must translate these into contextual norms, and then design requirements and thereby inform the design, development, implementation, and assessment of drones in the domain.

## Translating Ethical Principles into Values

### Beneficence

The first ethical principle, *beneficence,* states that biotechnology and AI should be “beneficial to humanity” (Floridi et al. 2018). Going further, it has been argued that there is a moral *obligation* to create beneficial technology: “we have a higher order moral obligation to innovate when it leads to moral progress” (van den Hoven 2013). Beneficence has an aspirational quality; “among all the possible technologies that we can spend money and energy on, which ones do we hold to be the most worthwhile?” (Stilgoe et al. 2013). A challenge to beneficence is that fear or misinformation surrounding a technology could lead to unnecessary underutilization, and thus represent an opportunity cost if its benefits are not fully realized (Floridi et al. 2018).

Beneficence features prominently in discussions of the opportunities afforded by drone technology within public healthcare—in fact, drones may be adopted due to their beneficial attributes. These benefits can be conceptualized on an individual basis, such as increasing the effectiveness with which an individual patient is diagnosed and treated, or in a broader societal context, such as reducing the use of antibiotics, leading to fewer drug-resistant bacteria and improving health outcomes on a national or international scale.

Based on the approaches within bioethics and AI, and emerging empirical evidence, beneficence within the healthcare drone domain can be translated into the values of human welfare (as well as the welfare of non-human animals), jobs and human skills, and environmental sustainability.

#### Human Welfare

Perhaps the most beneficial use of healthcare drones is in the promotion and preservation of human welfare. Human welfare can be further specified as physical welfare or health, psychological welfare such as mental wellbeing, and material welfare including economics or cost-savings (Friedman and Kahn 2003). Commercially-operated healthcare cargo drones are already being used in several locations around the world, including in Rwanda by Zipline and in Switzerland by Matternet (Scott and Scott 2017). This indicates that there may be a potential for benefits in human health and/or material welfare (profits or cost-savings). In Denmark, the HealthDrone project predicts it can save 26 million euros per year by implementing healthcare drones (Health_Drone 2019).

#### Jobs/Human Skills

Healthcare drones could contribute in a positive way to the current sweep of ‘transformative automation’ (Reif 2017). The benefits could be far-reaching, allowing people to develop high-tech skills such as programming, operating and maintaining a drone fleet, and reducing or eliminating ‘dull, dirty, and dangerous’ work (Clarke 2014b; Finn and Wright 2012).

#### Environmental Sustainability

Environmental sustainability can be framed in a number of ways, including anthropocentrism, zoocentrism, sentientism, biocentrism, and ecocentrism (Gjerris et al. 2013). This influences how the welfare of humans should be prioritized (or not) compared to that of animals, plants, or the ecosystem, and has an impact on how and why healthcare drones are utilized.

Healthcare drones could lead to increased environmental sustainability.

Life-cycle assessments of commercial drones (Neuberger 2017) and short-range commercial drone package delivery networks (Stolaroff et al. 2018) show that carefully-designed networks can have reduced impact over current transportation methods. In many applications, the drone can have a much smaller mass than a road vehicle, consuming considerably less energy (Stolaroff et al. 2018). The drone’s energy source is typically the most important factor in its environmental impact (Neuberger 2017). The smaller drones currently available use electric power and could be charged from renewable energy sources such as wind or solar if these are available, unlike many of the fossil-fuel driven vehicles they would replace.

### Non-maleficence

The ethical principle of *non-maleficence* means that the technology should ‘do-no-harm’. Non-maleficence means avoiding the creation of changes or of technologies that make things worse. In the context of experimental technology, we have “obligations to take away existing harm, or to prevent harm or risks that do not originate in the experiment” (van de Poel 2016). It has been noted that “though ‘do good’ (beneficence) and ‘do no harm’ (non-maleficence) seem logically equivalent…they represent distinct principles” (Floridi et al. 2018). The former refers to helpful actions, while the latter refers to the prohibition of causing harm (Gert et al. 2006).

Risks to privacy, security, the devaluation of human skills, and the need for capability caution have been identified conceptually and empirically within biotechnology and AI (Floridi et al. 2018). In the drone domain, privacy, safety, and economic impacts (i.e. job loss or changes in the workforce due to increasing levels of automation) are some of the most-often identified harms (Luppicini and So 2016) (Thomasen 2017). Therefore, non-maleficence encompasses the relevant human values of privacy, safety, security, calmness, jobs and human skills, and environmental sustainability. The approach of capability caution is introduced as a means to minimize these risk of harm.

#### Privacy

Privacy violations are already a well-documented risk of drone operations (Luppicini and So 2016). Many drone-related privacy issues are linked with the use of cameras or other sensors that can capture personal data. It has been found that the presence of drones, or the possibility that one could be watched by a drone, can lead to a “chilling effect” that limits freedom of expression and innovation (Clarke 2014a). The chilling effect has serious consequences when it comes to participation in political action: “democratic freedoms are undermined by the chilling of political speech” (Clarke 2014a). In the context of policing and security applications, “the deterrent effect on illegal behavior is likely to be far less than the chilling effect on lawful social, economic, cultural and political behaviors” (Clarke 2014a). Even drones without cameras can violate the *perception* of privacy, as many people believe that all drones carry cameras (Bajde et al. 2017b). And it appears likely that privacy violations and the objectification enabled by drone technology will be most felt by women (Thomasen 2017). Privacy-by-design guidelines have been proposed to minimize these risks (Cavoukian 2012). The European Union’s General Data Protection Regulation (GDPR) is a legal requirement within the EU (European_Union 2016), but can also serve as a useful reference for engineers and designers outside of Europe when considering privacy and drone design.

#### Safety

Drones are physical objects in the world, and therefore pose a safety risk to people on the ground, manned aircraft, and animals. Technology developers (Radi 2013a, b) (la Cour-Harbo 2017b) and legislators (SORA 2017) are highly focused on drone safety, and especially on preventing fatalities. They have adopted a quantitative approach called “equivalent level of safety”, or ELS, borrowing the safety norm from commercial aircraft operations as a benchmark for an acceptable level of fatalities (SORA 2017). ELS aims for less than one fatality every ten million hours of drone operation (SORA 2017). It should be noted that there are important differences in how this risk is accepted. In commercial aviation, passengers actively accept the risk when they board the flight. In drone crashes that strike people on the ground, there is usually no opportunity to accept the risk of the drone operation—a potential violation of the right to informed consent grounded in the principle of autonomy. Secondary accidents initiated by drone operations, such as drivers getting distracted by the drone and crashing, are usually not considered in existing analyses (la Cour-Harbo 2017a). The true reliability of drones in service is still hard to determine, and many assumptions need to be made to produce these fatality estimates (la Cour-Harbo 2017b). Flying drones in ‘safety corridors’ which avoid populated areas are expected to reduce the exposure of people on the ground to these safety risks.

#### Security

Security risks may be perpetrated intentionally, through negligence, or even laziness, and may made easier if adequate protections such as encryption and password protection are not in place. For example, a person may attempt to hack a drone as a means to hijack and crash it on purpose (Cawthorne and Wynsberghe 2019). It is conceivable that a healthcare drone could be used to deliver dangerous cargo, such as a bomb (Marcolini and Koettl 2018). A healthcare drone’s carrying capacity, along with other design features, as well as the trustworthiness of those that have access to it factor heavily in the opportunities for misuse. The drone may have a payload capacity of several kilograms, giving the ability to carry an explosive the size of the one detonated in Venezuela which injured seven soldiers (Marcolini and Koettl 2018). In addition, data security could become compromised with healthcare drones. The world’s largest drone manufacturer, DJI located in China, has already faced security concerns. In 2016 it was reported that the company shared customer data with Chinese authorities, and more recently the U.S. army has banned the drones as they posed ‘operational risks’ (BBC 2017).

The drone’s design can mitigate some of these risks by at least making it more difficult to place unauthorized cargo in the drone; for example, by minimizing the cargo volume to only the required dimensions, and requiring that only authorized cargo be inserted before the system will allow the drone to launch. Additionally, preventing the drone from flying in unauthorized locations, such as within 5 km of a public airport and 8 km from a military airport as required in Denmark (Trafikstyrelsen 2017), can be accomplished using ‘geo-fencing’ as implemented in some existing software (DJI 2019). A further step would be to detect the presence of people nearby (while maintaining their privacy) and prevent the drones from being operated within a certain stand-off distance from them, such as over 50 m to maintain a ‘comfortable’ distance (Bajde et al. 2017a).

#### Jobs/Human Skills

Healthcare drones will likely contribute to the increase in automation-related job-loss (Reif 2017) and a continued shift in the skills which humans develop. The new jobs created as work is automated by drones will not be equivalent in kind or quantity to those they displace. They will require different skills and pay different amounts, impacting material welfare. Jobs such as monitoring an autonomous drone operation where very little input is required of the ‘safety pilot’ could be considered dull. There may be many manual tasks such as loading and unloading medical samples from the drone, changing batteries etc. that will need to be performed by humans as they are costly to automate. Changes in a workforce are to be expected over time; the risk with drones, automation, and AI is the speed at which this change could occur and the quantity of jobs impacted. An often-cited Oxford study estimated that 47% of US jobs were at risk of being automated (Frey and Osborne 2013). This risk could be mitigated with government-sponsored re-training, and countries with public healthcare often have a welfare ‘safety net’ which ensures that people are supported when unemployed or transitioning to a new job.

#### Calmness

Calmness is a value that is often identified in the VSD literature (Friedman and Kahn 2003; Spiekermann and Pallas 2006). Healthcare drones could be a distracting visual presence and exhibit an annoying sound profile. The level of annoyance will likely differ based on context, be it in the city, the suburbs, the countryside, or over oceans and forests. Early experiments by NASA have shown that the noise profile of drones is different and more annoying than that of typical city noise from road vehicles (Christian and Cabell 2017). The links between high levels of noise pollution and negative impacts on human physical and psychological wellbeing have been well established (Passchier-Vermeer and Passchier 2000). Ideally, the drone should maintain calmness while still alerting people to its presence, so it is not thought to be a ‘spy’ drone. It should be noted that healthcare drones could increase calmness in some applications—for example, by replacing loud and disruptive medical helicopters.

#### Environmental Sustainability

Drones offer potential environmental benefits, but they also bring with them environmental risks. Drones are complex products which will become electronic waste or ‘e-waste’ at their end-of-life (EoL). Circular economy principles and design for EoL can help mitigate their impacts. Circular economy principles include the concepts of reuse, refurbishment, remanufacturing, and recycling, in order of decreasing efficiency (Parajuly 2017). Once healthcare drones reach their end-of-life, they could be reused within a similar context. If the drone is built in a modular fashion, components can easily be exchanged, repaired, and upgraded, and the drone can be customized to fit new uses. Drone power systems typically employ lithium-polymer batteries, which have a detrimental impact on human health during their production, but are recycled at a very high rate, at least in the European Union where a 95% rate is legislated (Notter et al. 2010). Drone structures are usually made of exotic materials such as carbon fiber reinforced epoxy which are non-recyclable and toxic to manufacture (CES 2019). Future drones may switch from battery power to fossil fuels for their increased energy density and range (Stolaroff 2014), releasing greenhouse gasses and particulates at their source. The mere presence of drones could lead to a ‘rebound effect’, where the (either perceived or real) efficiency of the drone leads to increased usage, offsetting or surpassing the environmental benefits (van den Hoven et al. 2015).

Healthcare drone operations could have a detrimental impacts on birds, bats, and other non-human animals. There is limited research on bird-drone interaction, but ethical guidelines for approaching birds with drones have been proposed (Vas et al. 2015), and ethical guidelines prohibit repeatedly exposing birds to the stress caused by drones (Lyons et al. 2018). Existing data indicate that most birds are visibly unaffected by the presence of a drone (Lyons et al. 2018), although stress cannot always be identified through visual means. Healthcare transportation drones may spend much of their time cruising at altitudes of up to 100 m; therefore, their interference with birds on or near the ground will be minimal, especially if the drones are not loitering (near nests, for example). However, hawks, eagles, and other birds of prey are territorial, soar at high altitudes, and have been known to attack multirotor and fixed-wing drones (Lyons et al. 2018). Logistics company Amazon is developing systems to prevent their delivery drones from hitting flocks of geese and other ‘non-collaborative flying objects’ (InsuranceJournal 2017). The impact of the drone’s sound on birds has not been extensively studied (Lyons et al. 2018). A multirotor drone’s sound profile was tested to see if it interfered with the echolocation of bats. It was found that the switching frequency of the electronic motor controllers did coincide with the bats echolocation frequencies, but that it could be shifted outside the bats audible range (Egeberg and Lundby 2017). In the future, ornithologists, chiropterologists and other experts can be consulted and represent the voice of impacted animals during the VSD process, such that the drone’s design and behavior minimize harm to these non-human stakeholders.

#### Capability Caution

Capability caution could be a useful approach in addressing non-maleficence in drone design. Capability caution refers to the need for careful definition of the upper limits of technological capabilities, and to developing and operating technology “within secure constraints” (Floridi et al. 2018). It could be argued that many risks of drones come from their possessing more capabilities than necessary to perform the task at hand, such as privacy risks resulting from unnecessary image collection (van Wynsberghe and Nagenborg 2016). Another capability risk arises when drones developed in a military context are applied to civil applications (sometimes called “dual use”), and vice versa (van Wynsberghe and Nagenborg 2016). Military drones possess capabilities and support values which are not relevant, or at least not as relevant, in civilian contexts, such as survivability (van Wynsberghe and Nagenborg 2016). Dual use can have important implications from a performance perspective as well, as the context of use is critical in the development of robust design requirements. Utilizing the approach of capability caution, each drone design would be developed for a more specific context of use. A ‘one-size-fits-all’ or ‘universal’ drone platform would not be possible as environments, stakeholders, values, and norms vary widely. A specific type of capability caution within the drone domain is privacy-by-design (Cavoukian 2012). Under privacy-by-design principles, drone operations should be geographically confined/geo-fenced (Blank et al. 2018; Cavoukian 2012). Anonymous video analytics software should be used to detect and destroy sensitive data, such as human faces and video frames containing them, in real-time, thus avoiding risks of privacy violations at the source (Cavoukian 2012).

Capability caution conflicts with economies-of-scale, which lead manufacturers to standardized and universal products. Additionally, capability caution means that manufacturers and designers have taken a more active role in specifying the conditions under which the technology is useful—its use plan (Vermaas et al. 2011). This has implications for responsibility: some use-plans are no longer possible as the designer has limited them and, through the product design, taken over some control from the user. When taken to the extreme, the result is technological paternalism where the will of users is subdued to that of the technology and the designer (Spiekermann and Pallas 2006). It should be noted, however, that designers, with their specialized knowledge, can and should in good faith create products which help people fulfill their version of the good life (Wong 2013).

### Autonomy

Autonomy here refers to respect for human autonomy (in contrast with the autonomy of a drone) and includes the free choice of individuals and groups (van de Poel 2016). In the context of drones used in public healthcare, the ethical principle of autonomy is translated into the human values of agency, responsibility, and trust (Floridi et al. 2018).

A critical element of human autonomy is informed consent, which is the idea that those exposed to a (new) technology should be made aware of its presence and must approve of its use (van de Poel 2016). VSD analyses have led to the use of informed consent in web applications (Friedman et al. 2000) and within the design of information and communications technologies (Spiekermann 2015). A similar approach may be appropriate with drone technologies. First, the public would be informed that a healthcare drone operation is planned, the reason for the operation, and the potential benefits and risks of the flight. Second, they would need to give their consent to allow the operation take place above them. This leads to the question of how many people, or if all of them, should give consent. One approach would be to establish a threshold—if, for example, a certain percentage of the population strongly disapprove of the operation (10% is identified as a heuristic for a ‘value dam’ in the literature Friedman and Hendry 2019), the operation would be halted. If informed consent was not obtained, this would need to be justified.

A challenge in implementing this approach is that it may be difficult to correctly identify all the relevant risks and benefits of new technologies (van de Poel 2016). Additionally, “it is sometimes questionable whether it is ethically desirable because it would give each individual that is affected a veto power however large the benefits to society” (van de Poel 2016). The inconvenience of needing to give consent must also be considered, but should not be considered a robust defense for bypassing consent. Traditionally, such healthcare projects move forward without explicit informed consent, as government officials and technology developers give consent-by-proxy when the project is deemed ready for use in the public space—usually, after testing in controlled environments where participants have given their consent to participate. Here, the default setting is critical: if the public is assumed to have ‘opted-in’ and accepted the operation, or ‘opted-out’ and rejected the operation.

#### Agency

“In very general terms, an agent is a being with the capacity to act, and ‘agency’ denotes the exercise or manifestation of this capacity” (Schlosser 2019). Human agency includes decision making and control, including control over autonomous systems like drones. Drones can possess varying levels of autonomy (not to be confused with human autonomy discussed earlier)—they can be fully autonomous or ‘human-out-of-the-loop’, human supervised or ‘human-on-the-loop’, or directly human operated, ‘human-in-the-loop’ (Clarke 2014b).^1^ The drone’s level and type of autonomy will have a direct impact on the ways in which human agency is (or is not) respected, and must be carefully designed rather than simply maximizing the drone’s autonomy to reduce the cost of human operators.

#### Responsibility

Responsibility includes praiseworthiness, blameworthiness, liability, and obligation (van de Poel and Sand 2018). Attributing responsibility for drone operations is challenging in practice as they are, by definition, remote and therefore take place at some distance from the operator. The EU legislation identifies two types of operations: those where the drone is within visual line of sight (VLOS) of the pilot and those that are beyond visual line of sight (BVLOS) of the operator (European_Union 2019). Currently, most operations are VLOS, with additional safeguards and restrictions on BVLOS drones and operations (European_Union 2019). Even in VLOS operations, the pilot can be up to a few hundred meters away which makes ascribing responsibility for the system and its behaviors difficult.

A relevant concept here is that of meaningful human control (MHC) of autonomous or semi-autonomous systems. MHC states that “humans not computers and their algorithms should ultimately remain in control of, and thus morally responsible for, relevant decisions…” (Santoni de Sio and van den Hoven 2018). It has been proposed that meaningful human control can be attained over (semi-)autonomous systems if two conditions are met. The first condition, called the tracking condition requires that the system:

should demonstrably and verifiably be responsive to the human moral reasons relevant in the circumstances—no matter how many system levels, models, software, or devices of whatever nature separate a human being from the ultimate effects in the world, some of which may be lethal. That is, decision-making systems should track (relevant) human moral reasons.

(Santoni de Sio and van den Hoven 2018).

The second condition, called the tracing condition requires that the system’s actions:

be traceable to a proper moral standing on the part of one or more relevant human persons who design or interact with the system, meaning that there is at least one human agent in the design history or use context involved in designing, programming, operating and deploying the autonomous system who (a) understands or is in the position to understand the capabilities of the system and the possible effects in the world of the its use; (b) understands or is in the position to understand that others may have legitimate moral reactions toward them because of how the system affects the world and the role they occupy.

(Santoni de Sio and van den Hoven 2018).

Thus, responsibility in healthcare drone design and operation can be reasonably assured if both conditions of MHC are met and incorporated into the system’s specifications. It has been proposed that MHC “can be one of the central notions of thinking about Responsible Innovation in robotics and AI” (Santoni de Sio and van den Hoven 2018).

#### Trust

Trusting technologies, organizations, or people requires that we can be vulnerable to them, that we think well of them, and that we are confident in their capabilities (McLeod 2015). Citizens that do not trust the government find healthcare drones, especially drones equipped with cameras, to be an additional intrusion (Bajde et al. 2017a; Scharf 2019). Yet, in general, healthcare drones are well positioned to be considered trustworthy (or at least more trustworthy than other types of drones) as they are seen as being used for a ‘good purpose’ (Bajde et al. 2017a). A risk of high levels of trust in the government and technology developers is that drones could be pushed onto the public in a paternalistic way (see the discussion about technological paternalism in the section on capability caution). This risk can be mitigated through the inclusion of a diverse set of impacted stakeholders (Friedman and Hendry 2019) and even critics (van de Poel 2000) during the design and implementation process—a key element in VSD. Including certain design features such as lights or markings which indicate the drone’s presence and mission can play heavily on enhancing the level of trust (as well as the related ethical principle of explicability discussed later). Location monitoring of healthcare drones (via GPS, for example) could have implications for employee trust by allowing oversight of their productivity, such as via the number of flights performed per day, as has been done with UPS delivery trucks in a bid to increase productivity (NPR 2014).

It has been shown that in some situations people have too high a degree of trust in robots and that people will follow the instructions of a robot in an emergency situation even when it is giving unsafe and unintuitive instructions (Robinette et al. 2016). A similar risk is possible with healthcare drones—that people assume the drone has capabilities such as sophisticated ‘sense and avoid’ that it may not possess. Using anthropomorphization is one strategy that has been used to gain the trust and acceptance of people in interactions with robots or drones, such as giving the robot a human or cartoon-like appearance or movements (Pakrasi et al. 2018). This seems reasonable in cases where it aids the explicability of the drone (discussed in more detail later), such as making it behave sluggishly when the battery is almost dead, but disingenuous when it is only to engender trust in a ‘cute’ drone.

### Justice

The ethical principle of justice includes obligations relating to issues of distributive justice, special protection of vulnerable groups, avoiding exploitation, and to just procedures (van de Poel 2016). It can be translated into the human value of fairness, including the equitable distribution of benefits and harms (Floridi et al. 2018).

#### Fairness

Who stands to benefit and who stands to be harmed by public healthcare drones, and are these benefits and risks equitably distributed? In broad terms, public healthcare drones could support the value of fairness—the distribution of benefits and risks could potentially be relatively evenly distributed throughout the population. Public healthcare is funded by taxpayers, and cost reductions or better healthcare outcomes achieved by adoption of drones will benefit everyone. The risks of drone operations, particularly safety and privacy, will also be somewhat distributed across the population as the drone flies overhead. This is in contrast to other drone operations which could have much clearer ‘winners’ and ‘losers’, such as a stealthy policing drone: the benefits are felt by the operators (the police), while the safety and privacy risks are placed on the public (van Wynsberghe and Nagenborg 2016). However, a more fine-grained analysis of each specific application area will reveal inequalities. For example, increased blood sample testing facilitated by drones will most directly benefit those over the age of 65—they are most at risk of diseases such as the flu that can be identified through these tests (Tillett et al. 1983). Residents living near drone launch and landing sites will be more at risk of drone-related accidents, visual, and noise pollution.

What about healthcare drones’ impact on vulnerable groups? “Aerial robots are ideal platforms for ‘individuals and groups seeking to impose their own morality on others’ (Novitzky et al. 2018)” (Clarke 2014b). This means that a range of actors with a range of objectives could use drones to facilitate their aims, including ‘legitimate’ actors such as some governments, or other powerful actors, such as private companies and technology developers. This risk is greatest when there is a large difference in power between actors, such as in humanitarian healthcare applications (Cawthorne and Cenci 2019).

The adoption of healthcare drones could lead to ‘technological lock-in’, where the technology justifies the reduction or divestment in infrastructure in local hospitals or clinics, reducing the availability of in-person care. In developing countries especially this could include reduced investment in infrastructure such as roads, bridges, and ferry routes, limiting peoples’ ability to move freely. However, healthcare drones could as well connect people in remote places (islands, for example) to modern healthcare, allowing them to live remotely but still be able to receive quality healthcare.

### Explicability

The final ethical principle, explicability, comes from the ethical framework for AI (Floridi et al. 2018). This principle refers to technological ‘transparency’, and was added due to the unique features of AI which can sometimes appear as a ‘black box’ (Sood 2018). Explicability deals with the ease at which systems can be understood. Within the context of drones for public healthcare, the principle of explicability can be translated into the values of intelligibility and accountability.

#### Intelligibility

Intelligibility in the epistemological sense is an answer to the question “how does it work?” (Floridi and Cowls 2019), or “what is happening inside the ‘black box’?” (van de Poel and Sand 2018). Healthcare drones present several challenges to intelligibility. When in operation they will, most of the time, be at a significant distance from the public and the pilot, making visible means of conveying information difficult. Drones are often small, again limiting visibility. They are often painted in basic colors such as black or white which gives no clue to their origins, and they often display flashing lights that are not standardized or easily understandable. The drone’s shape or silhouette may be critical to intelligibility, as it can be related with its function and is a feature that is visible from a distance.

The drone may have limited or no ability (i.e. sensors) to detect that there are people around it. This leads to a one-way type of human–robot interaction—the human *perception* of the drone. Alternatively, if the drone has sensors that can detect people, then a two-way, human-drone *interaction* can occur: the human perceives the drone and the drone (or drone operator) perceives the human. This allows for the drone to modify its behaviors around people, for example, moving away from those that show visible discomfort with its presence (i.e. staring at the drone, expressing a ‘negative stance’, and uttering negative emotions Bajde et al. 2017b). These improved sensing capabilities create challenges for privacy which much be mitigated, and if, for example, cameras are used, then it must be clearly indicated through the design (for example, via a ‘camera’ icon or a flashing red ‘record’ light). Other changes in drone design could also aid intelligibility; for example, fixed wing (aircraft-type) drones that are always flying forward make it easier to see the direction they are going compared to symmetrical multirotor (helicopter) drones. Colors, markings, and lights could be chosen that reflect the drone’s origins and purpose and would increase intelligibility to those noticing it. In the case of an emergency response drone, ambulance lights, colors, and markings could be used.

#### Accountability

Accountability is a response to the question “who is responsible for the way (the drone) works?” (Floridi and Cowls 2019). We differentiate responsibility (discussed earlier) with accountability—here we refer to how visible (explicable) the responsibility has been made. Accountability is a key and inherent challenge in the drone domain as operations are by their very nature remote. Accountability must be addressed for varying situations, including when personnel interact with the drone at the hospital, when members of the public see it during its operation, and those that find it if the drone ever crashes. Ideally, those who interact with the drone will be able to easily ascertain the identity of the pilot and the organization that is in responsible for it. Remote operations create power asymmetries—the drone may be able to see you, but, often, you can’t see who is behind the drone. A potential mitigation is to include the operator’s and/or owners name, picture, or identification number on the drone, or via a mobile app, but again, issues of operator privacy must also be addressed.

Experiments have shown that people expect every drone has one human operator, and that the operator is nearby; if they see a drone flying, they look around for the pilot (Bajde et al. 2017a, b).

Currently, in the EU legislation requires that one pilot has ultimate responsibility over a drone (European_Union 2019). This aids accountability since there is a one-to-one relationship between drone and pilot, and addresses the tracing requirement for meaningful human control (MHC) of autonomous systems (see the section on responsibility for more information on MHC). However, economic pressure and the potential of autonomous systems to increase safety over manual control could make it attractive to charge one pilot with oversight of multiple drones or a drone swarm, violating the tracing condition.

#### Meta-Explicability

The ethical framework itself can be used to address a meta-form of explicability—it can promote transparency by explicitly stating the ethical principles and human values that the drone’s design aims to support. The ethical principles, value conflicts and trade-offs, stakeholders, and so-on, can be made publicly available (via a website, for example), or even marked on the drone via an “ethical quality mark” (FRR 2019), “trust label” (Floridi et al. 2018), or ensured via an “ethics certification program” (IEEE 2019a).

## From Values to Norms and Design Requirements

The use of an ethical framework can facilitate the consideration of ethics and human values in technology design, and is especially useful as a practical, applied ethics tool for those with limited experience in technology ethics. As presented here, it is meant to facilitate and structure the decision making of engineers and designers without prescribing specific design choices. We have provided some discussion of relevant norms and a few design requirements for illustrative purposes throughout the paper, but the field is still new, and the contexts of use and impacted stakeholders vary so much that further specification should be performed by the relevant engineers and designers. In the future, when there are engineering standards in place after years of experience (the first standards are only being proposed now IEEE 2019b) the process will be more focused. Until then, we suggest that the ethical principles and human values we present via the framework can structure, in an iterative fashion, an ethically-informed process for determining design choices and requirements.

**Fig. 3:**
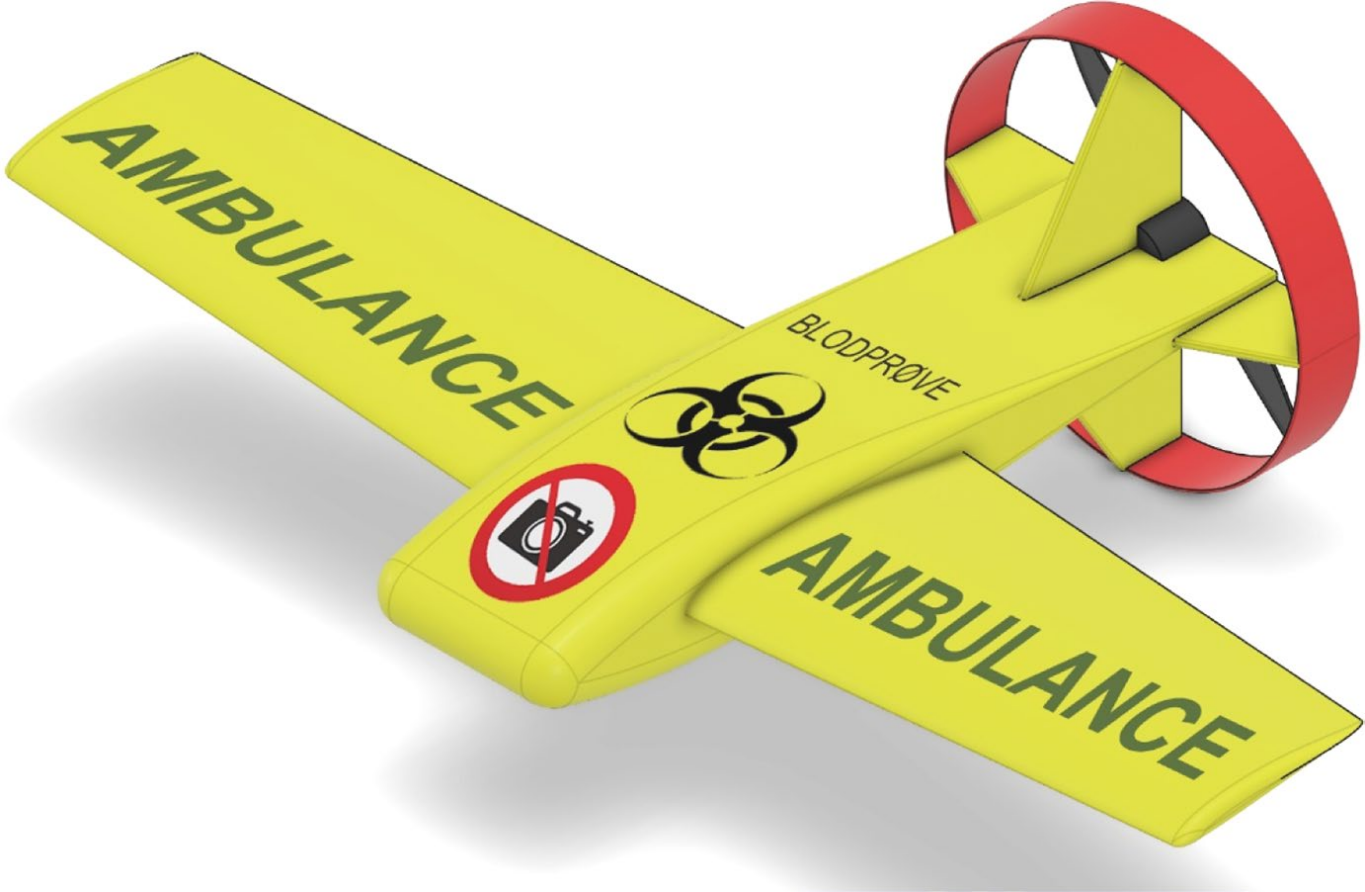
The ethical framework has been tested and refined via a case study where a drone was designed for blood sample transportation within Danish public healthcare (Cawthorne and Wynsberghe 2019)

This framework has been applied by the authors and used to develop a drone for blood sample transportation within Danish public healthcare (Cawthorne and Wynsberghe 2019). The drone’s design is shown in Fig. 3. The framework helped identify and enhance the drone’s potential benefits and mitigate the risks. It led to a focus on mitigating safety and privacy risks, and on enhancing explicability. For example, consideration of safety and capability caution lead to minimizing the size and weight of the drone to under 1.5 kg while still having a useful payload capacity. A fixed-wing aircraft configuration was chosen to support cost saving to taxpayers as this type of drone is the least expensive. This configuration aided explicability, as the drone always flies forward—demonstrating by its behavior that it has a purpose and is not loitering (as a multirotor drone is capable of). Explicability enhancements were identified as being important in the context of use since the drone would fly over the public to reach major hospitals. The drone’s color, shape, and markings were chosen to mimic that of an ambulance, and a mobile app which would allow citizens to be notified of the drone’s presence was proposed.

The framework helped to identify many value and design conflicts (van de Poel 2015). There, an innovation approach, which attempts to create new solutions to address ethical problems, was used to address the conflicts. This case study supports the approach in the current work and provides one example of how the framework can be applied in practice.

## Strengths and Limitations of the Framework

The use of a values hierarchy addresses some of the limitations of top-down approaches where “general precepts are ‘applied’ to particular cases” (Jacobs and Huldtgren 2018). General precepts are often so abstract as to be difficult to make actionable, as is required in applied ethics. In this work, the hierarchy links the abstract ethical principles with human values, and, via contextual norms, links these to design requirements through a process of translation (van de Poel 2013). It also addresses some limits of bottom-up approaches where specific cases are studied. Here, such detailed analysis can make it difficult to ascertain the far-reaching ethical importance at hand. As well, there is a risk of the naturalistic fallacy—conflating stakeholder values with good values. The values hierarchy addresses this shortcoming by allowing one to also work bottom-up, whereby design requirements are linked with more abstract values by ‘for the sake of’ relations (van de Poel 2013).

With any ethical framework there is a risk of overly-rigid application which could result in practitioners being blinded to emergent issues that do not fit within the five ethical principles or the listed values. Therefore, an abductive application of the framework is recommended: it should be used as a guideline for areas to investigate but used in conjunction with other VSD methods such as gathering stakeholder inputs. These inputs will either support or contradict the framework, leading to an ongoing process of co-creation and co-modification over time. This also addresses the change of human values over time (van de Poel 2018). For example, Van de Poel (2016) found no reference to the ethical principle of justice in the Nuremburg Code, but did so in the newer codes of ethics. Technological development is relevant as well—consider the addition of the principle of explicability made by Floridi et al. (2018) in view of the new capabilities afforded by AI.

## Conclusion and Future Work

From a societal perspective and on social timescales, the transition from a world without to a world with healthcare drones could be quite abrupt—with regards to jobs and the time required to retrain or educate people as drone operators. Therefore, combining the ethical framework, anticipatory methods, gradual introduction, and building in feedback mechanisms—so lessons can be learned from these experiments—could prove to be the best approach. This ensures that epistemological constraints are met, while also stressing “the role of uncertainty and ignorance, and the need for learning” (van de Poel 2016).

The merit of an ethical framework as an applied ethics tool rests on its ability to be used in practice. Therefore, the next-steps are to continue to apply and refine the framework, while developing drones within the domain—an ongoing process of co-creation and co-modification over time. The blood sample transportation drone shown in Fig. 3—an academic, public, and private collaborative effort—will be completed and tested. Coordination with legal bodies should take place, and careful consideration must be given as to if some elements of the framework should become legal requirements, such as how GDPR has become a legal requirement to protect privacy. The creation of this ethical framework reinforces the value of integrating ethics into practice and serves as a model for design and development in drone and non-drone domains. These tools could serve as helpful guides to designers and engineers, and facilitate the responsible design, development, implementation, and assessment of many types of technologies.

## Footnotes

1 See Cummings (2006) for a more detailed list of ten levels and types of autonomy with varying reliance on human inputs.
